# Translation and measurement properties of pregnancy and childbirth questionnaire in Iranian postpartum women

**DOI:** 10.1186/s12913-024-10689-7

**Published:** 2024-03-22

**Authors:** Somayeh Abdolalipour, Shamsi Abbasalizadeh, Sakineh Mohammad-Alizadeh-Charandabi, Fatemeh Abbasalizadeh, Shayesteh Jahanfar, Mohammad Asghari Jafarabadi, Kosar Abdollahi, Mojgan Mirghafourvadsnd

**Affiliations:** 1 Department of Midwifery, Faculty of Nursing and Midwifery, Tabriz University of Medical Sciences, Tabriz, IR Iran; 2 Women’s Reproductive Health Research Center, Tabriz University of Medical Sciences, Tabriz, Iran; 3 Department of Public Health and Community Medicine, Tufts School of Medicine, Boston, USA; 4 Cabrini Research, Cabrini Health, 3144 Melbourne, VIC Australia; 5 School of Public Health and Preventative Medicine, Faculty of Medicine, Nursing and Health Sciences, Monash University, 3800 Melbourne, VIC Australia; 6 Road Traffic Injury Research Center, Tabriz University of Medical Sciences, Tabriz, Iran; 7 Students Research Committee, Midwifery Department, Faculty of Nursing and Midwifery, Tabriz University of Medical Sciences, Tabriz, Iran; 8 Social determinants of Health Research Center, Tabriz University of Medical Sciences, Tabriz, IR Iran

**Keywords:** Quality of care, Prenatal care, Intrapartum care, Satisfaction, Women

## Abstract

**Background:**

Perceived care quality and patient satisfaction have been important care quality indicators in recent decades, and healthcare professionals have been influential on women’s childbirth experience. This study investigated the measurement properties of the Persian version of the Pregnancy and Childbirth Questionnaire (PCQ), designed to measure mothers’ satisfaction with the quality of healthcare services provided during pregnancy and childbirth.

**Methods:**

This is a cross-sectional methodological study. Instrument translation, face validity, content validity, structural validity, and reliability evaluation were performed to determine the measurement properties of the PCQ’s Persian version. A backward-forward approach was employed for the translation process. Impact scores were selected based on the items’ importance to measure face validity. Content validity index (CVI) and content validity ratio (CVR) were calculated to measure content validity, and exploratory and confirmatory factor analyses were used to measure structural validity. The cluster random sampling method was used, resulting in a sample of 250 eligible women referred to the health centers of Tabriz, Iran, who were 4 to 6 weeks after giving birth. Cronbach’s alpha coefficient and Intraclass Correlation Coefficient (ICC) using a test-retest approach were used to determine the questionnaire’s reliability.

**Results:**

The impact scores of all items were above 1.5, which indicates a suitable face validity. The content validity was also favorable (CVR = 0.95, CVI = 0.90). Exploratory factor analysis on 25 items led to the removal of item 2 due to a factor loading of less than 0.3 and the extraction of three factors explaining 65.07% of the variances. The results of the sample adequacy size were significant (< 0.001, and Kaiser-Meyer-Olkin = 0.886). The model’s validity was confirmed based on the confirmatory factor analysis fit indicators (i.e., RMSEA = 0.08, SRMR = 0.09, TLI = 0.91, CFI = 0.93, x^2^/df = 4.65). The tool’s reliability was also confirmed (Cronbach’s alpha = 0.88, and ICC (95% CI) = 0.93 (0.88 to 0.95)).

**Conclusion:**

The validity and reliability of the PCQ’s Persian version were suitable to measure the extent to which Iranian women are satisfied with the quality of prenatal and intrapartum care.

**Supplementary Information:**

The online version contains supplementary material available at 10.1186/s12913-024-10689-7.

## Background

Healthcare systems are primarily concerned with delivering effective evidence-based services to meet clients’ clinical/medical needs and their expectations of good quality care [1]. Therefore, in recent decades, perceived care quality and patient satisfaction have become important indicators of care quality [2]. Pregnant women often refer to different care providers, which may interfere with personal treatment and continuity of care and negatively affect women’s satisfaction with the care they receive [3]. Birth attendants’ continuous support during childbirth improves the childbirth experience [4].

Mother’s satisfaction and childbirth experience are essential factors with significant short-term and long-term consequences on mother and child, such as postpartum depression, post-traumatic stress disorder, breastfeeding ability, and child abuse. Healthcare professionals influence women’s childbirth experience [5].

Millions of women around the world still fail to access prenatal, intrapartum, and postpartum health services [6]. Many healthcare problems are due to poor quality of care [7]. The percentage of perinatal care quality in a study on pregnant women in Iran was as follows: 50.8% inadequate, 16.1% average, 27.7% adequate, and 5.4% excellent [8]. Therefore, it is necessary to analyze and monitor the quality of the care provided during pregnancy and childbirth.

Assessing the quality of care should be primarily based on the experience of the target group. Many tools are designed to examine women’s satisfaction with the care provided by health systems. For example, the questionnaire Measuring Satisfaction with Maternal and Newborn Health Care Following Childbirth, published in 2011 in the United States, was proposed to measure the mothers’ satisfaction with the postpartum health care provided to mothers and their babies until two months after childbirth [9].

The Quality of Prenatal Care Questionnaire (QPCQ) was designed in Canada in 2014 to measure the quality of prenatal care 4 to 6 weeks after childbirth [10]. The pregnancy- and maternity-care patients’ experiences questionnaire (PreMaPEQ) with 16 items and 145 items was designed in 2015 to measure the pregnancy, childbirth, and postpartum care, as well as public health clinics’ care provided to pregnant women in Norway. The questionnaire should be completed about 4 to 12 months after delivery. This relatively long period may affect the results’ accuracy during pregnancy and childbirth due to the memory limitations of the respondents [11]. In addition, the Measurement of Midwifery Quality Postpartum (MMAY postpartum) questionnaire with 16 items was designed in Germany in 2021 to measure the quality of midwifery care after childbirth from the mothers’ perspective. It measures only the quality of care provided at home until about four months after delivery [12].

Truijens et al. (2014) designed the Pregnancy and Childbirth Questionnaire (PCQ), which is a valid (good face validity and structural validity) and reliable (high internal consistency) instrument. This questionnaire has 25 items and measures Dutch mothers’ satisfaction with the quality of care provided during pregnancy in health centers and childbirth in hospitals [3]. The questionnaire should be filled out about 4–6 weeks after childbirth. Eighteen items examine the experiences and perceptions of pregnant women regarding the quality of prenatal care, divided into personal treatment (11 items) and educational information (7 items). The rest (7 items) reflect mothers’ satisfaction with intrapartum care.

The PCQ was prepared for countries where childbirth happens at home and in hospitals. Since the perceived quality of care is a general concept and is not limited to a specific care system, this questionnaire applies to any system. Measuring the quality of healthcare provided to pregnant women during pregnancy and childbirth and responding to their needs and expectations is increasingly essential. Therefore, this study was conducted to investigate the measurement properties of the PCQ as a valid and reliable tool to measure the quality of care provided to pregnant women in Iran during pregnancy and childbirth.

## Methods

### Study design

This cross-sectional methodological study follows five translation stages: content validity, face validity, structural validity, and reliability evaluation to determine the measurement characteristics of the PCQ’s Persian version. The target population includes pregnant women referring to health centers in Tabriz, Iran.

### Sample size

The structural validity in factor analysis requires at least 5 to 10 participants per the questionnaire’s items [13]. This study selects participants per each of the questionnaire’s 25 items, leading to a sample of 125 participants. Considering the design effect of 2 due to cluster sampling, the sample was calculated as 250.

### Eligibility criteria

Inclusion criteria include women who had vaginal childbirth in the last 4–6 weeks. The exclusion criteria include underlying diseases such as cardiovascular disease, diabetes, mental disabilities, or other mental disorders, the death of a loved one in the past three months, and the unwillingness to participate in the study.

### Sampling and data collection

We used *the*www.random.org website and randomly selected a quarter of the public health centers of Tabriz City from their list in the SIB system (https://sib.iums.ac.ir). The SIB system is an integrated health system and was designed to register, maintain, and update the electronic health record information of Iranians. Also, the type of health care services needed in community health centers are entered and recorded in this system. Then, we identified and called women who had given birth in the last 4 to 6 weeks. The research objectives were explained to them, and they consented to participate in face-to-face meetings in the health center at a given time. In the meetings, participants were informed comprehensively about the research, their written consent was obtained, and the researcher filled out the sociodemographic and obstetric characteristics questionnaire and the PCQ. Because some participants were illiterate or had low educational levels, to ensure the uniformity of the data collection method, the interview method was conducted to obtain the data. All interviews were conducted by a researcher (K.A.). Because only women with vaginal childbirth in the last 4 to 6 weeks were selected from the list in the SIB system (which was the main inclusion criterion for this study), the number of people who were excluded was small. Three people due to gestational hypertension, two people due to s diabetes mellitus, and six people due to unwillingness to participate in the study were not invited for the interview.

### The research tools

#### Sociodemographic and obstetric characteristics questionnaire

This questionnaire is a researcher-made tool that includes some questions used to describe participants’ characteristics such as age, education, occupation, income, number of pregnancies and deliveries, and participation in childbirth preparation classes. The validity of this questionnaire was measured through qualitative content and face validity.

#### The PCQ questionnaire

includes 25 items, of which 18 items measure the quality of perinatal care and seven measure the quality of childbirth care. The questionnaire uses a five-point Likert scale from completely agree (1) to completely disagree (5). The PCQ scores can vary from 25 to 125, with higher scores correlated with higher satisfaction levels [3]. The original version is available as a supplementary file 1.

### The translation processes

After obtaining permission from the initial designers of PCQ, Truijens et al. [3], the translation process was carried out using a five-step forward and backward translation approach [14]. First, the questionnaire items were translated separately by at least two translators fluent in Persian and English using semantic translation and a forward-translation approach. Contrary to literal translation, semantic translation transfers the essential meanings to the destination language. In other words, the translated questionnaire’s questions and words should be the same as those of the original. Second, the forward versions were compared by another supervisor translator, the existing contradictions were corrected, and a consolidated version was created from the forward versions. Third, the consolidated version was translated using a backward-translation approach into English by two translators fluent in Persian and English, who were blind to the questionnaires. Fourth, the expert committee, including three to four translators fluent in both languages, revised the forward, consolidated, and backward versions. The committee included one expert in language, one expert in questionnaire translation, one expert familiar with the concepts, and one coordinator. The experts investigated semantic, terminology, experimental, and perceptual equalities. The fifth stage includes the pre-test, where the pre-final version is provided to the target group.

### Face validity

Once the questionnaire’s final version was prepared, the face validity determination form was given to 10 women who had a delivery in the last 4–6 weeks. The items were evaluated in terms of difficulty, relevance, and ambiguity by ten eligible women to confirm the questionnaire’s qualitative face validity. In addition, the item impact method using a 5-point Likert scale from unimportant (1) to very important (5) was used to calculate the impact scores and confirm the questionnaire’s quantitative face validity, retaining items with an impact score of greater than 1.5 [15].

### Content validity

The content validity form was given to 10 midwifery and reproductive health specialists to check its content validity. The content validity of the tool was evaluated from a qualitative perspective based on the experts’ opinions on the questionnaire’s overall structure, the items’ contents, Persian grammar, and accurate scoring were received, and the necessary corrections were made. Moreover, the content validity index (CVI) and content validity ratio (CVR) were calculated in the quantitative part. The experts were asked to determine the items’ relevance, clarity, and simplicity using a 4-point Likert scale to calculate the CVI. The CVI varies between 0 and 1 [16]; items with CVI > 0.79 were kept, items with 0.79 > CVI > 0.70 were revised, and items with CVI < 0.70 were removed. The CVR was performed based on the experts’ opinions about each of the tool’s items using a 3-point Likert scale in terms of the necessity (i.e., necessary, useful but unnecessary, and unnecessary). According to the experts’ opinions and using the Lawshe table, items with CVR > 0.62 were kept, and the rest were removed [17].

### Structural validity

Evaluation of structural validity was done by exploratory factor analysis (EFA) and confirmatory factor analysis (CFA) [18]. Employing both exploratory and confirmatory factor analyses was rooted in the nature of our study’s theoretical framework. While our initial approach was to test a pre-specified model based on existing theories, we also recognized that the solidity of the underlying theoretical structure could benefit from a more exploratory examination. The exploratory factor analysis allowed us to explore potential underlying structures in an unbiased manner, given the evolving nature of the research domain and the potential for undiscovered dimensions. Subsequently, the confirmatory factor analysis aimed to validate and confirm the proposed model, aligning with established theories as much as possible [19]. The EFA with Kaiser-Meyer Olkin criteria and Bartlett’s Sphericity test using the principal component analysis method with Varimax rotation (direct noblemen) were used to examine structural validity and extract factors with a factor loading of more than 0.3 [20]. Moreover, CFA uses the fit indices to check the model fit. The desired fit indices and their acceptable values to confirm the model are as follows: root mean score error of approximation (RMSEA) < 0.08, standardized root mean square residual (SRMR) < 0.10, Baseline vs. Saturated (Chi^2^ /df) < 5, comparative fit index (CFI) > 0.90 and Tucker-Lewis Index (TLI) > 0.90 [21].

### Reliability

Test-retest reliability and internal consistency were used to determine the questionnaire’s reliability [22]. The questionnaire completion process involved two stages separated by a two-week interval, during which 30 eligible participants took part to assess retesting stability. Participants were conveniently sampled, and data for this stage were gathered through in-person interviews. Internal consistency was determined based on Cronbach’s alpha coefficient. An intra-class correlation coefficient (ICC) greater than 0.7 was considered favorable [23].

### Statistical analyses

SPSS Statistics 25 (IBM Corp, Armonk, NY, USA) and STATA 15 (Statcorp, College Station, Texas, USA) were used to analyze data. Descriptive statistics such as frequency (percentage) and mean (standard deviation) were used to describe the sociodemographic and obstetric characteristics data, which were normally distributed. Content validity using CVR and CVI, face validity using the impact score, structural validity using EFA and CFA, and the reliability of the research tool using Cronbach’s alpha coefficient and ICC were examined.

### Ethical considerations

The required permissions were first obtained from the Ethics Committee of Tabriz University of Medical Sciences (IR.TBZMED.REC.1401.093). All ethical principles, including obtaining the necessary permission from the initial designers of the tool, obtaining written informed consent from all the participants, ensuring the confidentiality of their information, and the freedom to exit the study, were observed throughout the research.

## Results

The cluster sampling method was used, and 250 postpartum women were studied from August to December 2022. The PCQ descriptive characteristics are given in Table 1.

**Table 1:** Sociodemographic characteristics of participants and scores of PCQ (*n* = 250)

Characteristics	Mean (SD)
**Age** (year)	28 (5.9)
**Gravidity**	1.8 (0.7)
**Parity**	1.5 (0.5)
**Education**	**Number (Percent)**
Illiterate & Primary	17 (6.8)
Secondary School	53 (21.2)
High School	23 (9.2)
Diploma*	80 (32)
University	77 (30.8)
**Occupation**	
Housewife	200 (80)
Employee	50 (20)
**Income**	
Not Adequate	20 (8)
Somewhat Adequate	188 (75.2)
Adequate	42 (16.8)
**PCQ scores**	**Mean (SD) Minimum - Maximum**
PCPT	42.3 (8.3) 22–55
PCEI	26.4 (6.2) 11–35
IPC	27 (6.2) 14–35
Total	95.7 (18.9) 57–125

SD: Standard Deviation; PCQ: Pregnancy and Childbirth Questionnaire; PCPT: Prenatal Care-Personal

Treatment; PCEI: Prenatal Care-Educational Information; IPC: Intrapartum Care

*End of high school education

In investigating the qualitative face validity, all the questionnaire items were described appropriately and without ambiguity or difficulty. All of them scored at least 1.5 when investigating the quantitative face validity. The CVI and CVR were equal to 0.9 and 0.95, respectively, which indicates the acceptability of the content validity. Table 2 presents the results of face and content validities.

**Table 2:** The results for the content and face validity of the Iranian version of PCQ (*n* = 10)

Item	CVI	CVR	Impact score
PCPT-1	0.80	0.90	1.90
PCPT-2	0.83	0.90	4.00
PCPT-3	0.90	1.00	3.20
PCPT-4	0.83	0.90	4.30
PCPT-5	0.67	0.70	3.20
PCPT-6	0.96	1.00	4.70
PCPT-7	0.70	1.00	3.20
PCPT-8	1.00	1.00	3.36
PCPT-9	0.96	1.00	2.40
PCPT-10	1.00	1.00	3.43
PCPT-11	0.96	1.00	3.28
PCEI-12	0.86	1.00	2.87
PCEI-13	0.96	1.00	3.36
PCEI-14	1.00	1.00	3.96
PCEI-15	1.00	1.00	2.16
PCEI-16	1.00	1.00	2.66
PCEI-17	0.86	0.90	3.36
PCEI-18	1.00	1.00	2.80
IPC-19	0.90	1.00	2.22
IPC-20	0.83	0.90	3.78
IPC-21	1.00	1.00	2.73
IPC-22	0.90	1.00	3.44
IPC-23	0.76	0.70	3.36
IPC-24	0.96	0.90	3.78
IPC-25	0.96	1.00	2.59

CVI: Content Validity Index; CVR: Content Validity Ratio; PCQ: Pregnancy and Childbirth Questionnaire; PCPT: Prenatal Care-Personal Treatment; PCEI: Prenatal Care-Educational Information; IPC: Intrapartum Care

**Fig. 1:**
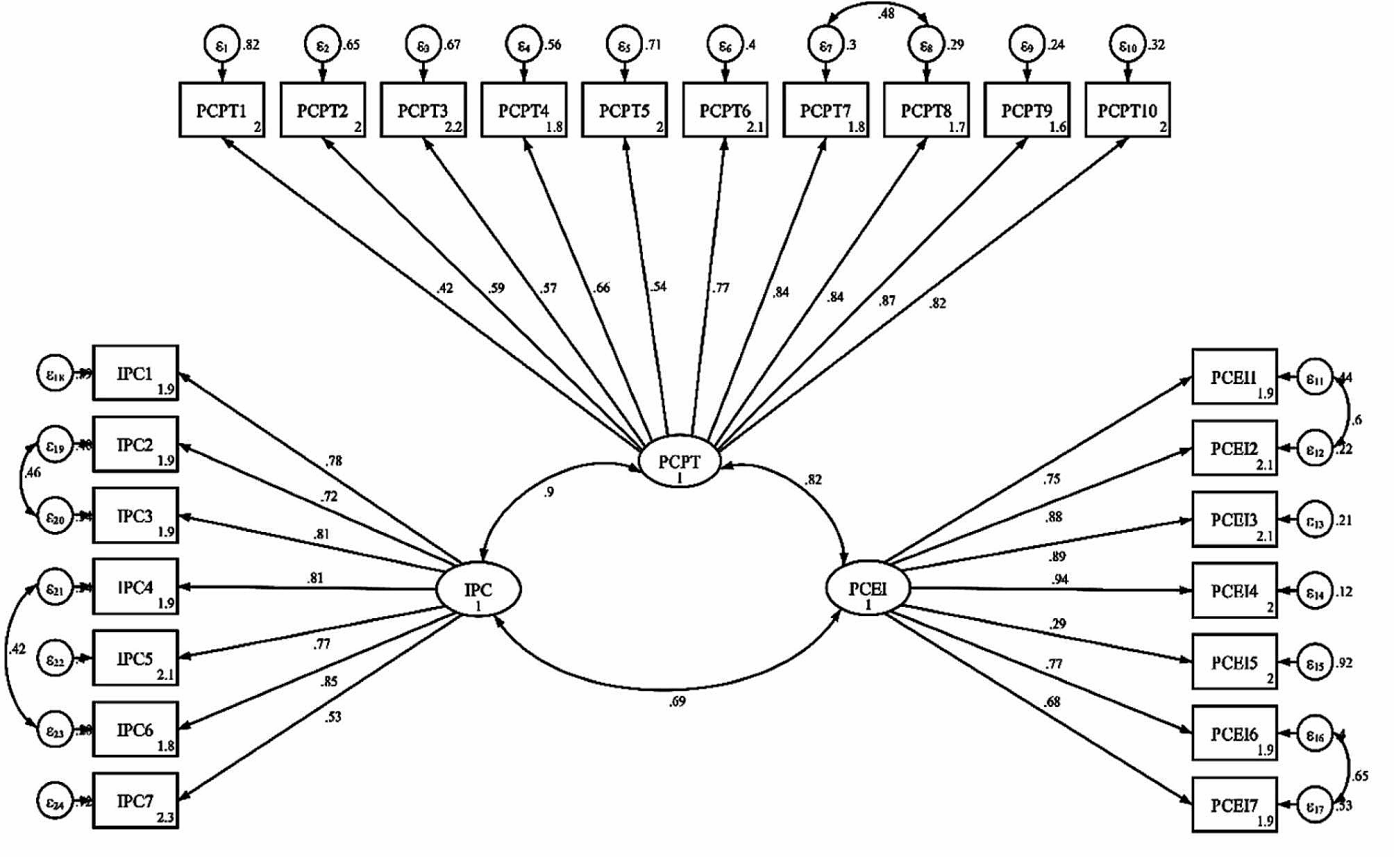
Factor structure model of the PCQ based on CFA. (All factor loadings are significant at *P* < 0.001). PCPT: Prenatal Care-Personal Treatment; PCEI: Prenatal Care Educational Information; IPC: Intrapartum Care

Three factors were extracted using the exploratory factor analysis on 25 items of the questionnaire. The first factor, i.e., prenatal care-personal treatment, included 11 items and explained 49.49% of the total variance. The second factor, i.e., prenatal care-educational information, included seven items and explained 10.98% of the total variance, and the third factor, i.e., intrapartum care, included eight items and accounted for 4.59% of the total variance (Table 3). Moreover, the second item during the exploratory factor analysis was removed due to its factor loading of less than 0.3, and the number of items was reduced from 25 to 24 (Fig. 1).

**Table 3:** Result of Facture analysis of the PCQ based on EFA (*n* = 250)

Items	Factor	Factor	Factor
	1	2	3
**Factor 1: Prenatal Care-Personal Treatment**			
Possibility to discuss things in confidence	0.431		
My partner was involved during prenatal visits	0.174		
Care provider was able to put my mind at ease	0.605		
I was involved in planning	0.586		
Treating personal information with confidence	0.672		
Sufficient amount of check-ups	0.594		
Communication between professionals	0.785		
Care providers aware of my preferences and wishes	0.819		
Clear who was in charge of care during pregnancy	0.843		
Treated in a respectful manner	0.857		
Participation in decision-making process	0.812		
**Factor 2: Prenatal Care– Educational Information**			
To discuss the pros and cons of screening		0.764	
Information regarding what to expect		0.902	
Information was complete		0.885	
Information satisfied my needs		0.923	
Quality of information can be improved		0.313	
Information regarding normal delivery		0.791	
Information regarding a healthy lifestyle		0.700	
**Factor 3: Intrapartum Care**			
Keeping informed on progress of birth			0.802
Paid attention to partner during delivery			0.782
Being aware of preferences and wishes			0.844
Communication with professionals during delivery			0.852
Communication between professionals			0.720
Clear who was in charge of care during delivery			0.856
Involved in decision making regarding anaesthesia			0.497
**% of variance** 49.5 11 5			
**Total score** 60.07			

PCQ: Pregnancy and Childbirth Questionnaire; EFA: Exploratory Factor Analysis

The KMO (0.886) was appropriate, indicating the adequacy of the sample size. The result of Bartlett’s Test of Sphericity (P˂ 0.001) was significant, indicating acceptable implementation of factor analysis concerning the correlation matrix on the study sample.

The results of CFA confirm a good fit of the model, and the model’s factor structure is confirmed (RMSEA (95% CI) = 0.081 (0.074 to 0.180), SRMR = 0.09, TLI = 0.91, CFI = 0.93, x^2^/df = 4.65, x2_ms = 1137.25, see Table 4).

**Table 4:** The model fit indicators of the PCQ (*n* = 250)

Goodness of fit indices	CFA	Acceptable value
**χ2**	1137.253	
$$ \raisebox{1ex}{${\varvec{x}}^{2}$}\!\left/ \!\raisebox{-1ex}{$\varvec{d}\varvec{f}$}\right.$$	4.659	< 5
**P-value**	< 0.001	0.05>
**CFI**	0.932	> 0.90
**TLI**	0.910	> 0.90
**SRMR**	0.091	< 0.10
**RMSEA (95% CI)**	0.081 (0.074–0.108)	< 0.08

χ2: chi-square; df: degrees of freedom; χ2/df: normed chi-square; CFI: Comparative Fit Index; TLI: Tucker–Lewis index; SRMR: Standardized root mean squared residual; RMSEA: root mean square error of approximation

Cronbach’s alpha was 0.88 for the whole tool, 0.78, 0.83, and 0.84 for the subdomains PCPT, PCEI, and IPC, in order. In addition, ICC (95% CI) for the whole tool was 0.93 (0.88 to 0.95) and 0.94 (0.90 to 0.96), 0.89 (0.83 to 0.93), and 0.86 (0.78 to 0.91) for above subdomains (Table 5). The final Persian version is available as a supplementary file 2.

**Table 5:** Reliability Statistics of the PCQ

Factors	Cronbach’s α coefficient	ICC (CI 95%) (*n* = 30)
PCPT	0.78	0.94 (0.90 to 0.96)
PCEI	0.83	0.89 (0.83 to 0.93)
IPC	0.84	0.86 (0.78 to 0.91)
PCQ (Total)	0.88	0.93 (0.83 to 0.93)

ICC: Intra-class Correlation Coefficient, CI: Confidence Interval, PCQ: Pregnancy and Childbirth Questionnaire; PCPT: Prenatal Care-Personal Treatment; PCEI: Prenatal Care Educational Information; IPC: Intrapartum Care

## Discussion

Women’s satisfaction with maternity services, especially care during childbirth and delivery, has become increasingly important to healthcare providers, managers, and policymakers, so increasing satisfaction is suggested to improve healthcare [24]. Measuring the quality of prenatal and intrapartum care is an essential step to evaluating its effectiveness more completely [10]. Therefore, appropriate measurement tools are needed to properly assess satisfaction with care during pregnancy and childbirth. This study investigates the measurement characteristics of the PCQ in Iranian women. The validity of this questionnaire was confirmed by face, content, and structural validity, and the reliability was also confirmed by test-retest and internal consistency in Iranian women.

Women who had received prenatal and intrapartum care completed the questionnaire, which is consistent with the growing consumer’s perspective in assessing healthcare quality. There are various maternity satisfaction measures, including single-item measures to extensive surveys of all aspects of maternity care [24–25].

The questionnaire’s items examine aspects such as communication, independence, participation, professionalism, educational information, teamwork, and spouse participation. Women, especially during childbirth, believe that the provided care should not be limited to providing information, but professionals with empathy and personal commitment should also understand their feelings and values [3, 26].

The questionnaire’s CVI and CVR were appropriate, and no item was removed. The model adequacy was confirmed using the value obtained for KMO and the significance of Bartlett’s test. Three factors similar to the original version were extracted for this tool, but the second item was removed from the subscale prenatal care-personal treatment. The removed item relates to the wife’s participation in prenatal care, which was removed due to its factor loading of less than 0.3. Moreover, the exploratory factor analysis extracted three factors, explaining 65.07% of the variance, which is higher compared to that of the original tool (56.2%) [3].

Cronbach’s alpha coefficient for the whole tool and subscales PCPT, PCEI, and IPC was equal to 0.88, 0.78, 0.83, and 0.84, respectively, which were acceptable, but compared to the original tool, with corresponding values of 0.92, 0.89, 0.83 and 0.86, were lower [3].

Many studies have examined satisfaction with maternal care, but there are a few valid tools with a particular focus on satisfaction during pregnancy and childbirth. The Labor and Delivery Satisfaction Index (LADSI) questionnaire with 38 items is frequently used to measure women’s satisfaction with prenatal and intrapartum care. However, the reliability of the whole tool (α = 0.34) and its subscales (i.e., caring component α = 0.11, and technical component α = 0.78) is low [27]. The Maternal Satisfaction for Caesarean Section (MSCS) questionnaire with 22 items has good validity and reliability but is limited to women giving birth by cesarean delivery [28]. Moreover, Intrapartal care concerning WHO recommendations (IC-WHO) questionnaire with 63 items was proposed to measure the quality of care during childbirth based on WHO recommendations [29]. Compared to other questionnaires, this tool focuses on women’s understanding of the care’s safety factor, and measurement is based on this. Intrapartal-Specific QPP-questionnaire (QPP-I) with 32 items asks women to evaluate the provided care during childbirth in terms of the perceived reality and the subjective importance of care [30]. This tool has good content and structure validity, but its reliability is reported to be low in some subscales (i.e., perceived reality subscales α range = 0.50 to 0.92; and subjective importance subscales α range = 0.49 to 0.93).

The pregnancy and maternity care patients’ experiences questionnaire (PreMaPEQ) is very comprehensive and measures women’s experiences during pregnancy, childbirth, and postpartum, as well as the care provided in public health clinics. Its content and structure validity are suitable, but its reliability in three scales was less than 0.7 [11]. This questionnaire has 145 items, which answers to them may be difficult for mothers. Moreover, the questionnaire is provided to women for completion about 4 to 12 months after childbirth, which limits its results’ accuracy due to possible memory limitations of mothers about their pregnancy and childbirth experiences. Some indications suggest that surveying time to measure satisfaction affects satisfaction ratings. For example, satisfaction with care can change even over a short period [24]. Moreover, women’s satisfaction at hospitals can significantly vary from those after discharge. Assessing satisfaction with childbirth at a certain time after childbirth seems to be more appropriate because mothers have enough time to review their experience and determine their satisfaction. However, the long periods may result in some biases in answering the questions. The PCQ is given to women about 4 to 6 weeks after childbirth, which seems a suitable time to measure mothers’ satisfaction with the quality of care during pregnancy and delivery.

### The research strengths and limitations

This study has some strengths including using the same data collection method (interview), a random selection of women from the health centers of Tabriz city with different socio-economic characteristics, and conducting interviews in the same time range (4 to 6 weeks) after childbirth. The studied women had only vaginal delivery and other childbirth types such as cesarean delivery were not included. Future research is suggested to apply this tool to women with cesarean delivery. Moreover, another research weakness was using the same set of data for exploratory and confirmatory factor analyses.

## Conclusion

The results confirmed the validity and reliability of the PCQ’s Persian version to measure the satisfaction level with the quality of prenatal and intrapartum care among Iranian women. Face, content, and structural validity and calculation of internal consistency and intra-class correlation coefficient were done for the assessment of measurement properties. This tool helps specialists and medical staff to evaluate the quality of care provided during pregnancy and childbirth from the women’s perspective and, if necessary, carry out the necessary interventions to improve the quality of the care.

### Electronic supplementary material

Below is the link to the electronic supplementary material.

Supplementary Material 1

## Data Availability

The datasets generated and/or analyzed during the current study are not publicly available due to the limitations of ethical approval involving the patient data and anonymity but are available from the corresponding author upon reasonable requests.

## Abbreviations

PCQ: Pregnancy and childbirth questionnaire
PCPT: Prenatal care-personal treatment
PCEI: Prenatal care educational information
IPC: Intrapartum care
EFA: Exploratory factor analysis
CFA: Confirmatory factor analysis
ICC: Intra-class Correlation Coefficient
SD: Standard deviation
CVI: Content validity index
CVR: Content validity ratio
df: Degree of freedom
KMO: Kaiser-Meyer-Olkin
RMSEA: Root mean squared error of approximation
CFI: Comparative fit index
TLI: Tucker–Lewis index

## References

[1] Kyei-Nimakoh M, Carolan-Olah M, McCann TV (2017). Access barriers to obstetric care at health facilities in sub-saharan Africa—a systematic review. Syst Reviews.

[2] Truijens SE, Banga FR, Fransen AF, Pop VJ, van Runnard Heimel PJ, Oei SG (2015). The effect of multi-professional simulation-based obstetric team training on patient-reported quality of care: a pilot study. Simul Healthc.

[3] Truijens SE, Pommer AM, van Runnard Heimel PJ, Verhoeven CJ, Oei SG, Pop VJ (2014). Development of the pregnancy and Childbirth Questionnaire (PCQ): evaluating quality of care as perceived by women who recently gave birth. Eur J Obstet Gynecol Reprod Biol.

[4] Perdok H, Verhoeven CJ, Van Dillen J, Schuitmaker TJ, Hoogendoorn K, Colli J, Schellevis FG, De Jonge A (2018). Continuity of care is an important and distinct aspect of childbirth experience: findings of a survey evaluating experienced continuity of care, experienced quality of care and women’s perception of labor. BMC Pregnancy Childbirth.

[5] Lemmens SM, van Montfort P, Meertens LJ, Spaanderman ME, Smits LJ, de Vries RG, Scheepers HC (2021). Perinatal factors related to pregnancy and childbirth satisfaction: a prospective cohort study. J Psychosom Obstet Gynecol.

[6] Khayat S, Dolatian M, Navidian A, Mahmoodi Z (2018). Factors Affecting Adequacy of Prenatal Care in Suburban women of Southeast Iran: A Cross -sectional study. J Clin Diagn Res.

[7] Bahmani S, Shahoie R, Rahmani K (2022). The quality of prenatal care from the perspective of the service recipients using the Servqual pattern during the COVID-19 pandemic in Sanandaj Comprehensive Health Centers. Nurs Midwife J.

[8] Simbar M, Nahidi F, Akbarzadeh A (2012). Assessment of quality of prenatal care in Shahid Beheshti University of Medical Sciences health centers. Payesh J.

[9] Camacho FT, Weisman CS, Anderson RT, Hillemeier MM, Schaefer EW, Paul IM (2012). Development and validation of a scale measuring satisfaction with maternal and newborn health care following childbirth. Matern Child Health J.

[10] Heaman MI, Sword WA, Akhtar-Danesh N, Bradford A, Tough S, Janssen PA, Young DC, Kingston DA, Hutton EK, Helewa ME (2014). Quality of prenatal care questionnaire: instrument development and testing. BMC Pregnancy Childbirth.

[11] Sjetne IS, Iversen HH, Kjøllesdal JG (2015). A questionnaire to measure women’s experiences with pregnancy, birth, and postnatal care: instrument development and assessment following a national survey in Norway. BMC Pregnancy Childbirth.

[12] Peters M, Kolip P, Schäfers R (2021). A questionnaire to measure the quality of midwifery care in the postpartum period from women’s point of view: development and psychometric testing of MMAY postpartum. BMC Pregnancy Childbirth.

[13] Comrey A, Lee H (2013). A first course in factor analysis: psychology press.

[14] Lee WL, Chinna K (2019). The forward-backward and dual-panel translation methods are comparable in producing semantic equivalent versions of a heart quality of life questionnaire. Int J Nurs Pract.

[15] Setia MS (2017). Methodology Series Module 9: Designing questionnaires and Clinical Record forms - Part II. Indian J Dermatol.

[16] Polit DF, Beck CT, Owen SV (2007). Is the CVI an acceptable indicator of content validity? Appraisal and recommendations. Res Nurs Health.

[17] Lawshe CH (1975). A quantitative approach to content validity. Pers Psychol.

[18] Mokkink LB, Terwee CB, Knol DL, Stratford PW, Alonso J, Patrick DL, Bouter LM, de Vet HC (2010). The COSMIN checklist for evaluating the methodological quality of studies on measurement properties: a clarification of its content. BMC Med Res Methodol.

[19] Manapat PD, Anderson SF, Edwards MC. Evaluating avoidable heterogeneity in exploratory factor analysis results. Psychol Methods. 2023 May;11. 10.1037/met0000589. 10.1037/met0000589 37166853

[20] Harerimana A, Mtshali NG (2020). Using exploratory and Confirmatory Factor Analysis to understand the role of technology in nursing education. Nurse Educ Today.

[21] Schreiber J, Nora A, Stage F, Barlow L, King J. Confirmatory factor analyses and structural equations modeling: an introduction and review. J Educ Res. 2006;99(6).

[22] Rousson V, Gasser T, Seifert B (2002). Assessing interarater, interrater and test–retest reliability of continuous measurements. Stat Med.

[23] Cicchetti DV (1994). Guidelines, criteria, and rules of thumb for evaluating normed and standardized assessment instruments in psychology. Psychol Assess.

[24] Sawyer A, Ayers S, Abbott J, Gyte G, Rabe H, Duley L (2013). Measures of satisfaction with care during labor and birth: a comparative review. BMC Pregnancy Childbirth.

[25] Britton J (2012). The assessment of satisfaction with care in the perinatal period. J Psychosom Obstet Gynecol.

[26] Goberna-Tricas J, Banu´ s-Gime´nez MR, Palacio-Tauste A (2011). Satisfaction with pregnancy and birth services: the quality of maternity care services as experienced by women. Midwifery.

[27] Lomas J, Dore S, Enkin M, Mitchell A (1987). The labor and delivery satisfaction index– the development and evaluation of a soft outcome measure. Birth.

[28] Morgan PJ, Halpern S, Lo J (1999). The development of a maternal satisfaction scale for cesarean section. Int J Obstet Anesth.

[29] Sandin-Bojo AK, Larsson BW, Hall-Lord ML (2008). Women’s perception of intrapartal care in relation to WHO recommendations. J Clin Nurs.

[30] Wilde Larsson B, Larsson G, Kvist LJ, Sandin-Bojo AK (2010). Women’s opinions on intrapartal care: development of a theory-based questionnaire. J Clin Nurs.

