# Supplementary material for: Translation and measurement properties of pregnancy and childbirth questionnaire in Iranian postpartum women
Source: BMC Health Serv Res. 2024 Mar 22;24:365. doi: 10.1186/s12913-024-10689-7 (PMC10958905; doi:10.1186/s12913-024-10689-7)
Supplement: Supplementary file 1 — Supplementary Material 1 [file 12913_2024_10689_MOESM1_ESM.docx]

**Supplementary file 1:** The English version of the Pregnancy and Childbirth Questionnaire

| **Pregnancy and Childbirth Questionnaire (PCQ)** | **Totally agree** | **Somewhat agree** | **No idea** | **Somewhat disagree** | **Totally disagree** |
| --- | --- | --- | --- | --- | --- |
| **A. The quality of care during pregnancy**  **A1) Prenatal Care - Personal Treatment (11 items)** | | | | | |
| 1. Possibility to discuss things in confidence |  |  |  |  |  |
| 2. My partner was involved during prenatal visits |  |  |  |  |  |
| 3. Care provider was able to put my mind at ease |  |  |  |  |  |
| 4. I was involved in planning |  |  |  |  |  |
| 5. Treating personal information with confidence |  |  |  |  |  |
| 6. Sufficient amount of check-ups |  |  |  |  |  |
| 7. Communication between professionals |  |  |  |  |  |
| 8. Care providers aware of my preferences and wishes |  |  |  |  |  |
| 9. Clear who was in charge of care during pregnancy |  |  |  |  |  |
| 10. Treated in a respectful manner |  |  |  |  |  |
| 11. Participation in decision making process |  |  |  |  |  |
| **A2) Prenatal Care - Educational Information (7 items)** | | | | | |
| 12. To discuss the pros and cons of screening |  |  |  |  |  |
| 13. Information regarding what to expect |  |  |  |  |  |
| 14. Information was complete |  |  |  |  |  |
| 15. Information satisfied my needs |  |  |  |  |  |
| 16. Quality of information can be improved |  |  |  |  |  |
| 17. Information regarding normal delivery |  |  |  |  |  |
| 18. Information regarding a healthy lifestyle |  |  |  |  |  |
| **B) The quality of care during chldbirth (7 items)** | | | | | |
| 19. Keeping informed on progress of birth |  |  |  |  |  |
| 20. Paid attention to partner during delivery |  |  |  |  |  |
| 21. Being aware of preferences and wishes |  |  |  |  |  |
| 22. Communication with professionals during delivery |  |  |  |  |  |
| 23. Communication between professionals |  |  |  |  |  |
| 24. Clear who was in charge of care during delivery |  |  |  |  |  |
| 25. Involved in decision making regarding anesthesia |  |  |  |  |  |
